# Directed acoustic shadow enhancement for pre-incision ultrasound localization and precise incision planning in multiple rib fractures undergoing SSRF: a retrospective comparative feasibility study

**DOI:** 10.3389/fmed.2026.1851418

**Published:** 2026-05-25

**Authors:** Xueyi He, Pinhua Chen, Zhengchao Zhang, Ruoli Wang, Qi Fang, Yiren Zhu, Zhixian Xu, Wubing He

**Affiliations:** 1 Fuzhou University Affiliated Provincial Hospital, Fuzhou, Fujian, China; 2 Shengli Clinical Medical College of Fujian Medical University, Fuzhou, Fujian, China; 3 Department of Emergency Trauma Surgery, Fujian Provincial Hospital, Fuzhou, Fujian, China; 4 Fujian Trauma Medicine Center, Fuzhou, Fujian, China; 5 Fujian Key Laboratory of Emergency Medicine, Fuzhou, Fujian, China

**Keywords:** acoustic shadow, feasibility study, historical control, learning curve, rib fractures, surgical stabilization of rib fractures, ultrasound localization

## Abstract

**Background:**

Accurate pre-incision localization of fracture segments is essential for targeted exposure during surgical stabilization of rib fractures (SSRF). We evaluated Directed Acoustic Shadow Enhancement (DASE), a simple ultrasound-assisted localization technique using an external cotton swab as an acoustic reference marker to translate sonographic fracture findings into skin surface marking.

**Methods:**

This single-center retrospective comparative study included 91 adult patients with multiple (≥3) rib fractures who underwent SSRF, including 22 consecutive DASE-assisted cases and 69 historical controls treated using conventional preoperative localization. Both groups received the same intermuscular approach and nitinol memory alloy encircling fixation, with the principal difference being the pre-incision localization strategy. The primary outcomes were skin-to-skin operative time and incision length per plated rib. Secondary outcomes included blood loss, prophylactic intraoperative chest tube placement, postoperative day 3 visual analog scale (VAS) pain score, and length of stay. Atelectasis and pulmonary infection were analyzed as trauma-related preoperative thoracic complications rather than postoperative outcome events. The primary analytic framework consisted of full-cohort comparison and multivariable regression.

**Results:**

All 22 DASE cases achieved successful pre-incision localization without localization-related complications or conversion to conventional localization. Compared with historical controls, the DASE group had shorter operative time (78.5 ± 31.9 vs. 102.8 ± 31.9 min; *p* = 0.004), shorter total incision length (8.0 ± 3.0 vs. 10.7 ± 3.7 cm; *p* = 0.001), and shorter incision length per plated rib (2.53 ± 1.02 vs. 3.20 ± 1.25 cm/rib; *p* = 0.016). In multivariable regression, DASE remained associated with shorter operative time (adjusted *β*, −20.64 min; 95% CI, −37.75 to −3.52; *p* = 0.018), shorter incision length per plated rib (adjusted *β*, −0.70 cm/rib; 95% CI, −1.28 to −0.12; *p* = 0.018), and lower blood loss (adjusted *β*, −61.13 mL; 95% CI, −107.65 to −14.60; *p* = 0.010), although blood loss was interpreted cautiously. Case-sequential localization time in the DASE cohort showed a significant downward trend, suggesting a descriptive pattern consistent with an early learning phase.

**Conclusion:**

DASE appears to be a feasible and safe pre-incision ultrasound-assisted localization adjunct for SSRF. This technique may support more targeted exposure and operative planning; however, these findings should be interpreted cautiously and require confirmation in larger prospective studies.

## Introduction

Multiple rib fractures are common manifestations of blunt thoracic trauma and are associated with substantial morbidity, particularly when fracture burden is high, fracture displacement is pronounced, or chest wall mechanics are impaired (1, 2). In patients with flail chest or selected multiple rib fracture patterns, surgical stabilization of rib fractures (SSRF) has increasingly been adopted as a treatment strategy to improve pain control, facilitate respiratory recovery, and reduce pulmonary morbidity, although the magnitude of benefit varies across patient populations and study designs (3–6). As indications for SSRF have expanded, increasing attention has been directed toward operative strategies that minimize soft tissue disruption while maintaining accurate fracture targeting and stable fixation (7–9).

A key technical challenge in SSRF is accurate localization of the target fracture segments before incision. Preoperative computed tomography (CT), preferably with three-dimensional reconstruction, provides essential anatomic information for fracture selection and operative planning (7, 10). However, translating radiologic findings into precise skin-surface marking remains difficult in practice, particularly in posterior, lateral, or multi-segment fracture patterns. Even CT may miss subtle or nondisplaced rib fractures, and in limited-exposure SSRF, imprecise localization can lead to longer exploration, wider dissection, and less efficient access to the intended fracture sites (11). Thus, a practical adjunct that improves fracture-to-skin translation without adding radiation may be valuable during pre-incision planning.

Ultrasound has emerged as a useful tool for rib fracture assessment because it can directly demonstrate cortical disruption and associated sonographic abnormalities without ionizing radiation. A systematic review by Battle et al. suggested that ultrasound may be superior to chest radiography for detecting rib fractures after blunt chest wall trauma (12). More recently, Vassalou et al. reported that thoracic ultrasonography appeared superior to X-ray when CT was used as the reference standard (13). In a prospective emergency department study, Celik et al. found that ultrasonography showed high sensitivity for detecting rib fractures in patients with blunt chest trauma (14), and Zarei Jelyani et al. likewise reported clinically useful diagnostic performance for point-of-care ultrasound compared with CT, while also highlighting the influence of fracture burden and examination conditions on performance (15). Together, these studies support the potential role of ultrasound not only in diagnosis but also in operative planning.

The practical problem in operative planning, however, is not merely identifying a fracture on the screen, but converting that sonographic finding into an accurate and reproducible skin-surface mark that can guide incision design. This translational step is especially relevant when the operative objective is targeted exposure through an intermuscular approach. Prior work by Martin et al. suggested that perioperative ultrasound localization may help optimize the surgical approach for SSRF by reducing incision length and operative time (16). Nevertheless, ultrasound-guided localization in rib fixation remains a relatively underdeveloped technical domain, and current guideline-level discussion remains cautious, framing ultrasound as a potentially helpful adjunct rather than a definitive standard (1).

Directed Acoustic Shadow Enhancement (DASE) was developed to address this translational problem. By placing a cotton swab on the skin as an external reference marker and aligning its generated acoustic shadow with the sonographically identified fracture site, DASE is intended to map subsurface fracture findings onto the skin surface in a simple, low-cost, and reproducible manner. Because it uses standard ultrasound equipment and a straightforward external marker, DASE is best conceptualized as a practical operative-planning adjunct rather than a specialized imaging platform. The present study evaluated the initial clinical use of DASE in patients undergoing SSRF for multiple rib fractures. Rather than attempting to establish definitive superiority, we designed this study to assess the technical feasibility of DASE and to explore whether its use was associated with more targeted incision planning and favorable perioperative process measures when compared with historical controls treated using conventional localization strategies. We additionally assessed case-sequential localization time in the DASE cohort to describe the early learning pattern of the technique.

## Materials and methods

**Figure 1:**
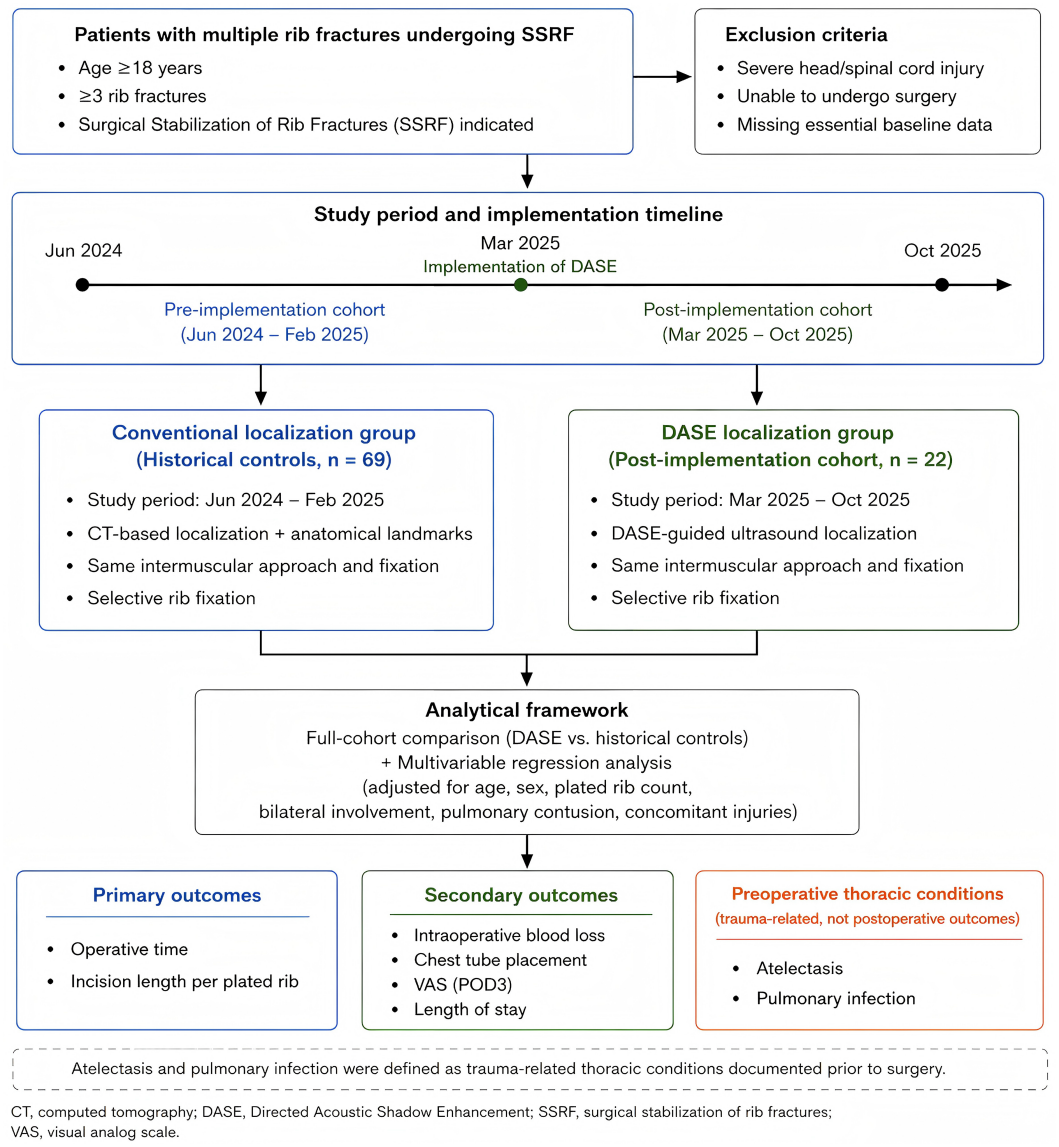
Study design and analytical framework. Flow diagram of the study. Adult patients with multiple (≥3) rib fractures who underwent surgical stabilization of rib fractures (SSRF) were retrospectively reviewed. The final analytic cohort included 22 DASE-assisted cases and 69 historical controls. The primary analytic framework consisted of full-cohort comparison and multivariable regression. Primary outcomes were operative time and incision length per plated rib. Atelectasis and pulmonary infection were analyzed as trauma-related preoperative thoracic complications rather than postoperative outcome events.

This was a single-center retrospective comparative study using a historical control design. Patients were consecutively identified from June 2024 to October 2025. The control cohort included patients treated between June 2024 and February 2025 using conventional localization, whereas the DASE cohort comprised patients treated between March 2025 and October 2025 following the implementation of DASE. The final analytic dataset included 91 patients with multiple rib fractures who underwent SSRF, including 22 consecutive patients who underwent pre-incision DASE-assisted ultrasound localization and 69 historical controls who underwent conventional preoperative localization (Figure 1).

Both groups underwent the same intermuscular (muscle-sparing) surgical approach and nitinol memory alloy encircling fixation. The principal difference between groups was the pre-incision localization strategy. Rib fixation was performed selectively based on surgical indication and fracture characteristics; not all radiographically identified fractures were plated, particularly in cases with bilateral or extensive fracture patterns. Inclusion criteria were patients aged ≥18 years with multiple (≥3) rib fractures undergoing SSRF and with complete perioperative records. Exclusion criteria included missing key perioperative outcome data or incomplete baseline clinical information, as well as patients unable to undergo surgery or with severe head or spinal cord injury. In the conventional group, preoperative localization was performed based on CT imaging findings, anatomical landmarks, and intraoperative palpation, with incision planning guided by surgeon experience.

**Figure 2:**
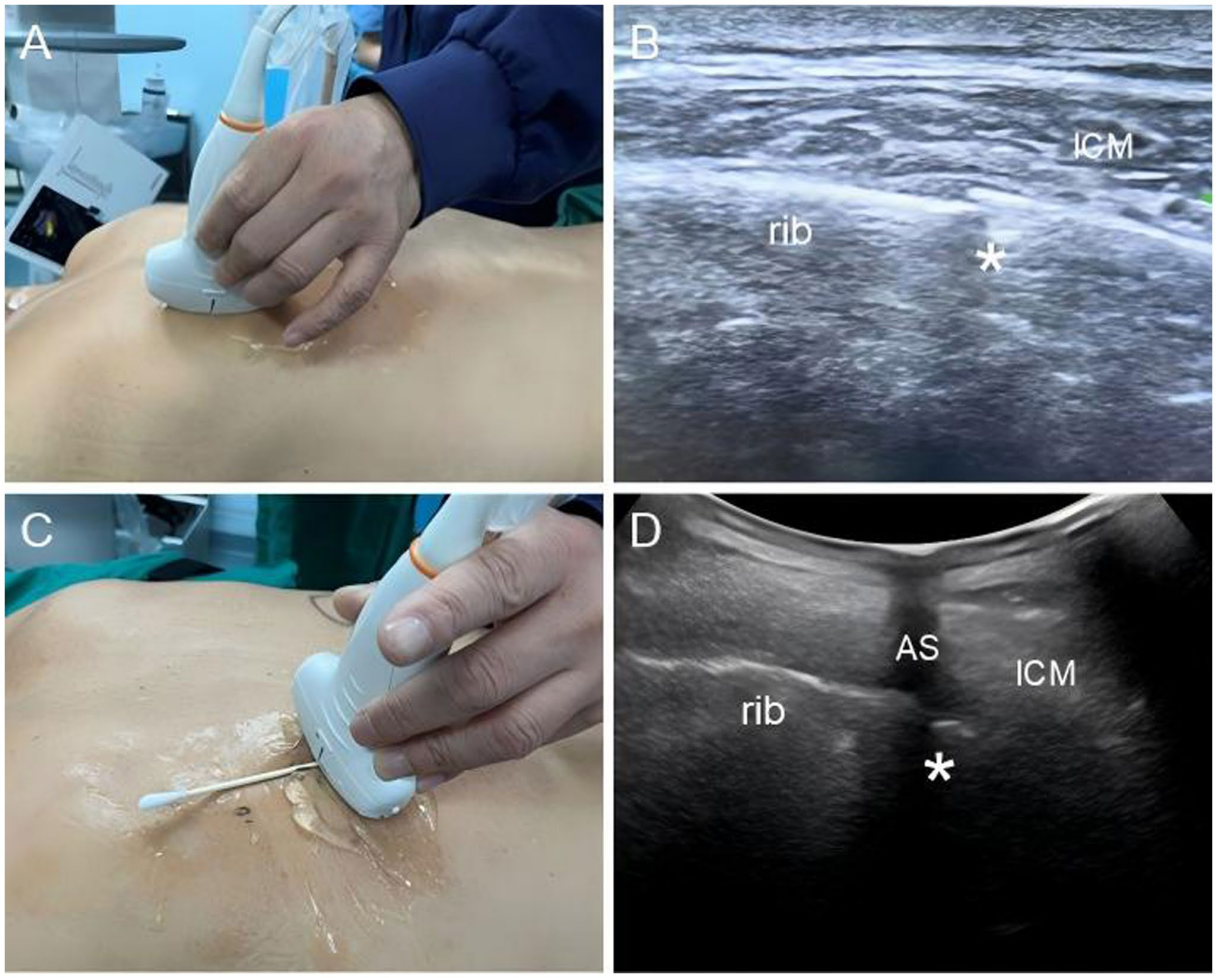
DASE technique for pre-incision rib localization. **(A)** Pre-incision ultrasound scanning along the rib surface. **(B)** Sonographic identification of a rib fracture, shown as cortical disruption of the rib surface with an associated acoustic shadow; the asterisk indicates the fracture site. **(C)** Placement of a cotton swab on the skin beneath the probe to generate an external acoustic reference shadow. **(D)** Alignment of the externally generated acoustic shadow with the identified fracture site to translate the subsurface finding into a skin-surface mark for incision planning. ICM, intercostal muscle; AS, acoustic shadow.

DASE was performed immediately before skin incision by the operating surgeon after basic ultrasound training. A portable ultrasound system with a high-frequency linear transducer was used for most cases, with a convex transducer selected for deeper or posterior rib segments when necessary. After the approximate fracture region had been identified based on preoperative imaging, the rib surface was scanned longitudinally. Rib fractures were recognized as cortical discontinuity or step-off deformity of the hyperechoic rib cortex. A cotton swab was then placed on the skin beneath the probe to generate an external acoustic shadow. By fine adjustment of the swab position and probe angle, the externally generated shadow was aligned with the sonographically identified fracture point, thereby translating the subsurface finding into an accurate skin-surface mark for incision planning (Figure 2).

The primary outcomes were skin-to-skin operative time and incision length per plated rib, calculated as total incision length divided by the number of plated ribs. Secondary outcomes included blood loss, prophylactic intraoperative chest tube placement, postoperative day 3 visual analog scale pain score, and length of stay. Technical feasibility outcomes in the DASE cohort included localization success, need for conversion to conventional localization, and localization-related complications.

Atelectasis and pulmonary infection were defined as trauma-related thoracic conditions documented prior to surgery based on admission imaging and clinical records rather than postoperative outcome events and were therefore analyzed as baseline characteristics. During data cleaning, fracture laterality codes 2 and 3, both used in different phases of data entry to indicate bilateral involvement, were harmonized as bilateral.

**Figure 3:**
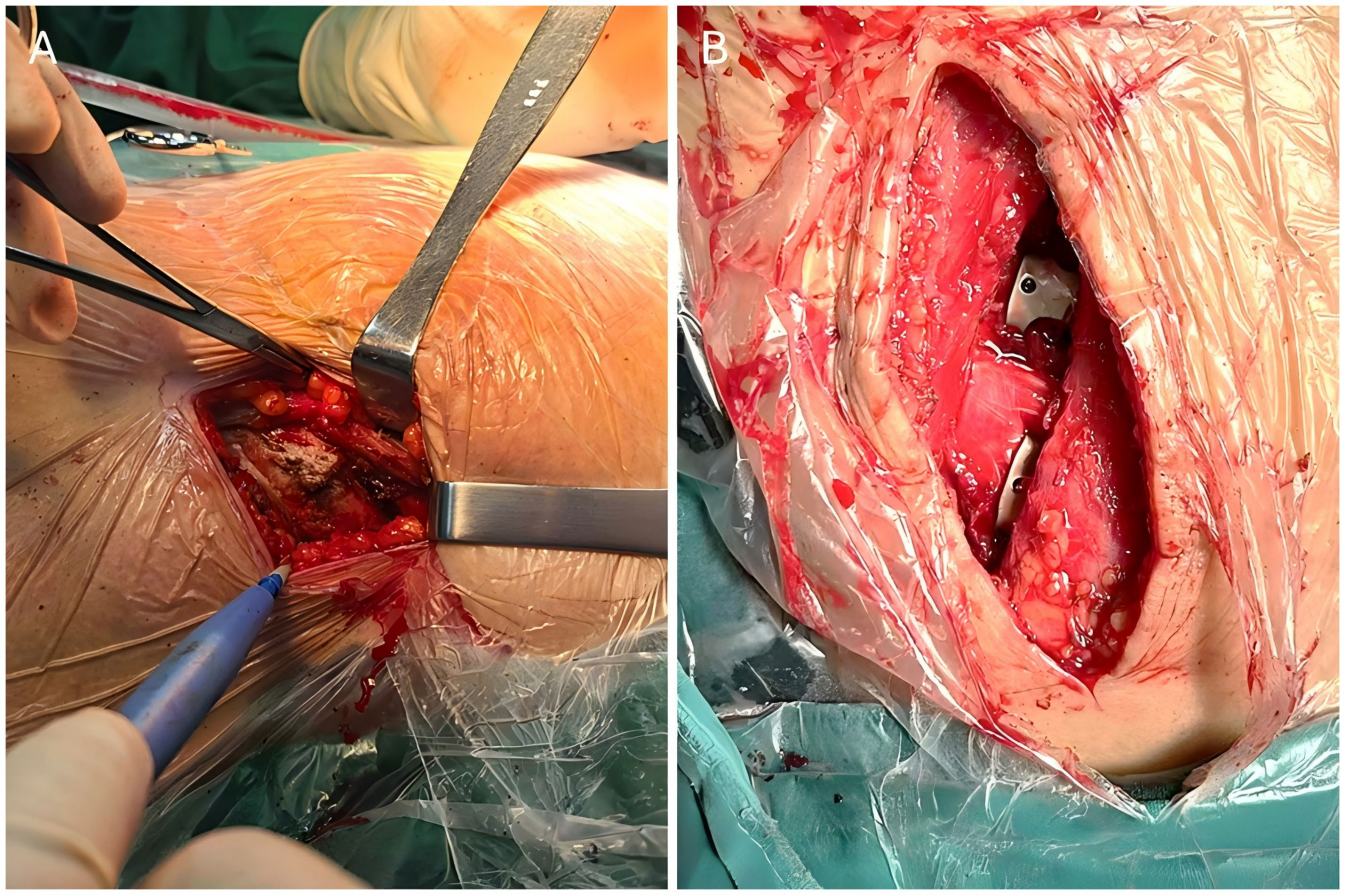
Intraoperative findings after DASE-guided planning. **(A)** Direct exposure of the targeted rib fracture through a limited intermuscular incision planned according to pre-incision DASE localization. **(B)** Stabilization of the fractured rib using a nitinol memory alloy encircling fixation device through the same intermuscular approach.

Continuous variables were summarized as mean ± standard deviation or median [interquartile range], as appropriate, and categorical variables as number (%). Between-group comparisons were performed using Welch *t* tests or Mann–Whitney U tests for continuous variables and Fisher exact tests or chi-square tests for categorical variables, as appropriate. The primary analytic framework consisted of full-cohort comparison and multivariable linear regression with robust standard errors. Prespecified covariates included age, sex, plated rib count, bilateral fracture involvement, pulmonary contusion, and concomitant injuries. Blood loss and length of stay were interpreted cautiously because they may also reflect overall trauma burden and, in some cases, concomitant procedures performed during the same operative session. Within the DASE cohort, case order was defined by the chronological order of the recorded cases, and the learning curve was described using case-sequential localization time and a 3-case moving average. A two-sided *p* value <0.05 was considered statistically significant (Figure 3).

## Results

The final analytic dataset comprised 22 DASE cases and 69 historical controls. Baseline characteristics are shown in Table 1. Compared with controls, the DASE cohort had a higher fractured rib count (8.1 ± 3.1 vs. 5.5 ± 2.0; *p* < 0.001), a higher proportion of bilateral fractures (50.0% vs. 13.0%; *p* = 0.001), and more concomitant injuries (54.5% vs. 26.1%; *p* = 0.019). Pulmonary contusion was less frequent in the DASE cohort (72.7% vs. 97.1%; *p* = 0.002), whereas preoperative pulmonary infection was more common (63.6% vs. 26.1%; *p* = 0.002). Plated rib count was similar between groups (3.5 ± 1.4 vs. 3.5 ± 1.2; *p* = 0.807).

**Table 1:** Baseline characteristics of the full cohort.

Variable	DASE (*n* = 22)	Historical controls (*n* = 69)	*P* value
Age, years	54.5 ± 12.3	52.1 ± 10.6	0.431
Male sex, *n* (%)	13 (59.1)	49 (71.0)	0.306
Mechanism of injury, *n* (%)			0.031
Motor vehicle accident	11 (50.0)	46 (66.7)	
Fall	7 (31.8)	6 (8.7)	
High fall	2 (9.1)	14 (20.3)	
Other	2 (9.1)	3 (4.3)	
Fractured ribs, *n*	8.1 ± 3.1	5.5 ± 2.0	<0.001
Plated ribs, *n*	3.5 ± 1.4	3.5 ± 1.2	0.807
Fracture laterality, *n* (%)			0.001
Left-sided	6 (27.3)	31 (44.9)	
Right-sided	5 (22.7)	29 (42.0)	
Bilateral	11 (50.0)	9 (13.0)	
Pulmonary contusion, *n* (%)	16 (72.7)	67 (97.1)	0.002
Preoperative atelectasis, *n* (%)	8 (36.4)	12 (17.4)	0.078
Preoperative pulmonary infection, *n* (%)	14 (63.6)	18 (26.1)	0.002
Concomitant injuries, *n* (%)	12 (54.5)	18 (26.1)	0.019

Data are presented as mean ± standard deviation or number (%), as appropriate. *p* values were calculated using Welch *t* test for continuous variables and chi-square test or Fisher exact test for categorical variables, as appropriate. For multi-category variables, the reported *p* value represents the overall between-group comparison. Atelectasis and pulmonary infection were analyzed as trauma-related preoperative thoracic complications rather than postoperative outcome events. Bilateral fracture involvement included cases coded as bilateral during data harmonization.

All 22 DASE cases achieved successful pre-incision localization without localization-related complications or conversion to conventional localization, supporting the technical feasibility of the workflow.

Unadjusted perioperative outcomes are summarized in Table 2. Compared with historical controls, the DASE cohort showed shorter operative time (78.5 ± 31.9 vs. 102.8 ± 31.9 min; *p* = 0.004), shorter total incision length (8.0 ± 3.0 vs. 10.7 ± 3.7 cm; *p* = 0.001), and shorter incision length per plated rib (2.53 ± 1.02 vs. 3.20 ± 1.25 cm/rib; *p* = 0.016). Prophylactic intraoperative chest tube placement was also less frequent in the DASE cohort (4.5% vs. 55.1%; *p* < 0.001). Blood loss differed between groups (50.0 [20.0–50.0] vs. 50.0 [30.0–100.0] mL; *p* = 0.008), but this variable was interpreted cautiously because it may have been influenced by operative complexity beyond localization strategy alone. Postoperative day 3 pain scores were similar (1.5 ± 0.7 vs. 1.3 ± 0.7; *p* = 0.131). Length of stay was longer in the DASE cohort in unadjusted comparison (20.5 [14.2–25.0] vs. 14.0 [10.0–19.0] days; *p* = 0.031).

**Table 2:** Unadjusted perioperative outcomes in the full cohort.

Outcome	DASE (*n* = 22)	Historical controls (*n* = 69)	*P* value
Operative time, min	78.5 ± 31.9	102.8 ± 31.9	0.004
Blood loss, mL	50.0 [20.0–50.0]	50.0 [30.0–100.0]	0.008
Total incision length, cm	8.0 ± 3.0	10.7 ± 3.7	0.001
Incision length per plated rib, cm/rib	2.53 ± 1.02	3.20 ± 1.25	0.016
Prophylactic intraoperative chest tube placement, *n* (%)	1 (4.5)	38 (55.1)	<0.001
Postoperative day 3 VAS score	1.5 ± 0.7	1.3 ± 0.7	0.131
Length of stay, d	20.5 [14.2–25.0]	14.0 [10.0–19.0]	0.031

Data are presented as mean ± standard deviation, median [interquartile range], or number (%), as appropriate. *P* values were calculated using Welch *t* test or Mann–Whitney U test for continuous variables and chi-square test or Fisher exact test for categorical variables, as appropriate. Blood loss and length of stay should be interpreted cautiously because they may also reflect overall trauma burden and, in some cases, concomitant procedures performed during the same operative session.

Multivariable regression results are presented in Table 3. After adjustment for age, sex, plated rib count, bilateral fracture involvement, pulmonary contusion, and concomitant injuries, DASE remained associated with shorter operative time (adjusted *β*, −20.64 min; 95% CI, −37.75 to −3.52; *p* = 0.018), shorter incision length per plated rib (adjusted *β*, −0.70 cm/rib; 95% CI, −1.28 to −0.12; *p* = 0.018), and lower blood loss (adjusted *β*, −61.13 mL; 95% CI, −107.65 to −14.60; *p* = 0.010). No statistically significant adjusted association was observed for postoperative day 3 pain score (adjusted *β*, 0.09; 95% CI, −0.28 to 0.46; *p* = 0.647) or length of stay (adjusted *β*, 2.79 days; 95% CI, −1.53 to 7.12; *p* = 0.206).

**Table 3:** Adjusted associations between DASE use and key perioperative outcomes.

Outcome	Adjusted *β* for DASE	95% CI	*P* value
Operative time, min	−20.64	−37.75 to −3.52	0.018
Incision length per plated rib, cm/rib	−0.70	−1.28 to −0.12	0.018
Blood loss, mL	−61.13	−107.65 to −14.60	0.010
Postoperative day 3 VAS score	0.09	−0.28 to 0.46	0.647
Length of stay, d	2.79	−1.53 to 7.12	0.206

Adjusted associations were estimated using multivariable linear regression with robust standard errors. DASE was entered as the exposure variable, with the historical control group as the reference. Models were adjusted for age, sex, plated rib count, bilateral fracture involvement, pulmonary contusion, and concomitant injuries. *β* coefficients represent adjusted mean differences with 95% confidence intervals. The prespecified adjusted incision metric was incision length per plated rib. Blood loss and length of stay were interpreted cautiously because they may not solely reflect the effect of localization strategy. Adjusted modeling for prophylactic intraoperative chest tube placement was not presented because this management-dependent outcome was interpreted as exploratory and the DASE group had a sparse event count.

**Figure 4:**
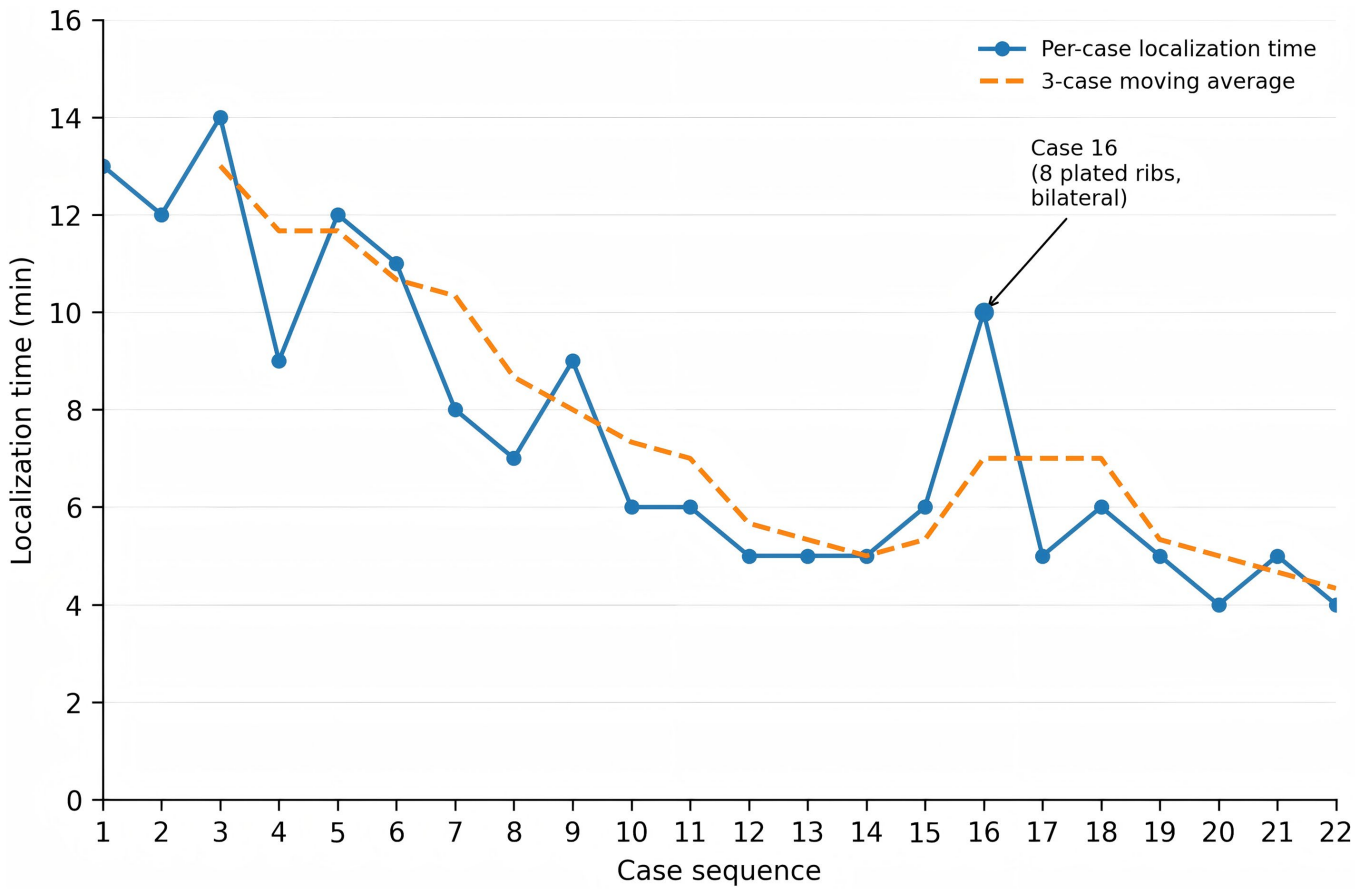
Learning curve of DASE-assisted localization. Case-sequential localization time across the 22 DASE cases is shown together with a 3-case moving average. Localization time decreased progressively with case sequence and then stabilized after the initial cases, suggesting a descriptive pattern consistent with an early learning phase. The transient rise at case 16 corresponded to bilateral fixation involving eight plated ribs.

Case-sequential localization time in the DASE cohort averaged 7.6 ± 3.1 min. The mean localization time was 9.7 ± 2.8 min in the first 11 cases and 5.5 ± 1.6 min in the second 11 cases. Figure 4 shows a marked downward trend in localization time with increasing case sequence, with relative stabilization after the initial cases and a transient rise at case 16, which involved bilateral fixation of eight plated ribs. Spearman analysis demonstrated a strong inverse association between case order and localization time (*ρ* = −0.85, *p* < 0.001), suggesting a descriptive pattern consistent with an early learning phase.

## Discussion

In this retrospective comparative study, DASE proved technically feasible as a pre-incision ultrasound-assisted localization strategy for patients undergoing SSRF for multiple rib fractures. All DASE cases were successfully localized without localization-related complications or conversion to conventional localization, supporting the practical operability of this workflow in routine surgical settings. In the comparative analysis, DASE use was associated with shorter operative time and shorter incision length per plated rib, and these associations remained present after multivariable adjustment. Taken together, these findings suggest that DASE may help improve the precision and efficiency of pre-incision planning, while the overall interpretation of outcome differences should remain cautious because of the study design and sample size.

The principal value of DASE lies in facilitating translation of sonographic fracture findings into actionable skin-surface marking for incision planning. In SSRF, successful exposure depends not only on identifying the relevant fracture targets radiographically, but also on converting imaging information into a limited and appropriately positioned incision. DASE was specifically developed to address this gap by using a simple external acoustic reference marker to align the sonographically identified fracture site with the skin surface. In this respect, DASE should be viewed less as a diagnostic innovation and more as a practical operative-planning adjunct. This framing is consistent with the current literature and with the more cautious evidentiary posture recommended by recent consensus work (1).

Our findings should also be interpreted in the context of prior ultrasound-assisted rib localization studies. Martin et al. reported that perioperative ultrasound localization during SSRF was associated with reduced incision length and operative time (16). Accordingly, the present study does not establish an entirely new conceptual role for ultrasound in rib fixation; rather, it explores a specific technical refinement in which an externally generated acoustic shadow is deliberately aligned with the fracture site to facilitate skin marking and incision planning. In that sense, DASE is better understood as a pragmatic extension of previously described ultrasound-guided localization strategies than as a replacement for CT-based preoperative assessment or other established planning tools.

The observed associations with shorter operative time and shorter incision length per plated rib are mechanistically plausible. More accurate pre-incision localization may reduce unnecessary exploration, facilitate direct access to the intended fracture zone, and limit soft tissue dissection during the formal operative phase (17). This interpretation is also consistent with the broader minimally invasive SSRF literature, which emphasizes that the benefits of limited-exposure fixation depend heavily on precise preoperative planning and accurate fracture targeting (7, 10, 18). In other words, DASE may not change the fixation principles themselves, but may help surgeons execute a targeted intermuscular approach more efficiently.

Blood loss was also lower after multivariable adjustment, but this result should be interpreted cautiously. In this retrospective setting, blood loss may not reflect the isolated effect of localization strategy alone, because it can also be influenced by global trauma burden, operative complexity, fracture distribution, and concomitant procedures performed during the same operative session.

Similarly, the marked difference in prophylactic intraoperative chest tube placement should be interpreted strictly as a management-related and exploratory finding rather than a direct effect of the intervention. Chest tube decisions are influenced by surgeon preference, intraoperative judgment, and evolving perioperative protocols and therefore cannot be causally attributed to the localization strategy. This difference may also reflect underlying differences in baseline thoracic pathology and injury severity between groups rather than the effect of the localization strategy itself.

It is also important to distinguish baseline thoracic complications from postoperative outcome events in this study. Atelectasis and pulmonary infection were analyzed as trauma-related preoperative thoracic complications rather than postoperative complications caused by surgery. Their higher frequency in the DASE cohort primarily reflects greater baseline injury complexity and likely helps explain why unadjusted length of stay was longer in the DASE cohort despite more favorable operative process measures. This distinction is important for avoiding over-interpretation of length-of-stay differences in a non-randomized dataset. This difference may also reflect underlying differences in baseline thoracic pathology and injury severity between groups rather than the effect of the localization strategy itself.

From a practical perspective, DASE may represent a relatively low-threshold localization method, but it is not entirely free from operator dependence. Ultrasound-based rib localization requires familiarity with chest wall sonoanatomy, recognition of cortical disruption and acoustic shadowing, and the ability to maintain probe-marker alignment while adapting to different fracture locations and patient positions (19, 20). Prior diagnostic studies have shown encouraging performance for ultrasound in rib fracture detection (12–14), but they also make clear that performance depends on user experience and examination context (21). In the present study, DASE was performed by the operating surgeon after basic ultrasound training, and case-sequential localization time showed a marked downward trend with subsequent stabilization. This pattern suggests that the core workflow may be learned during an early implementation phase, but formal learning-curve, reproducibility, and competency-threshold studies are still needed before broader dissemination can be recommended.

This study has several limitations. It was a single-center retrospective study using historical controls, and the DASE sample size remained limited. Residual confounding cannot be fully excluded even with multivariable adjustment, particularly because fracture burden, bilateral involvement, pulmonary status, and concomitant injuries differed between groups. In addition, some perioperative variables, especially blood loss and length of stay, may reflect overall trauma complexity and concomitant procedures rather than localization strategy alone. The learning-curve analysis was informative but descriptive, and it was not designed as a formal training study. Finally, because all patients still underwent standard CT-based preoperative evaluation, the present study does not support replacing CT with ultrasound; rather, it supports studying DASE as a supplementary pre-incision planning tool.

Because this study used a historical control design with consecutive time periods, potential era effects must be considered. The control cohort (June 2024 to February 2025) preceded the implementation of DASE, whereas the DASE cohort (March 2025 to October 2025) reflects a later phase of institutional experience. Although the same surgical team and operative approach were used, temporal changes in surgical workflow, perioperative management, and operator experience may have influenced the observed outcomes. Therefore, the findings should not be interpreted as reflecting the effect of the intervention alone.

Despite these limitations, the present study provides an initial clinical framework for understanding how a simple pre-incision ultrasound localization technique may support targeted SSRF exposure. Rather than claiming definitive outcome superiority, our data suggest that DASE is feasible and may improve operative efficiency and incision precision in selected patients. Future prospective studies should further evaluate localization accuracy, inter-operator reproducibility, implementation requirements, and patient-centered outcomes, ideally with standardized recording of training exposure, localization time, and operative decision-making variables.

## Conclusion

DASE appears to be a feasible and safe pre-incision ultrasound-assisted localization adjunct for patients undergoing SSRF for multiple rib fractures. By helping translate sonographic fracture findings into skin-surface marking, DASE may support more targeted exposure and shorter operative time. These findings should be regarded as exploratory and warrant confirmation in larger prospective studies.

## Data Availability

The original contributions presented in the study are included in the article/supplementary material, further inquiries can be directed to the corresponding author.
